# Supplementary material for: Modeling glioblastoma heterogeneity as a dynamic network of cell states
Source: Mol Syst Biol. 2021 Sep 16;17(9):e10105. doi: 10.15252/msb.202010105 (PMC8444284; doi:10.15252/msb.202010105)
Supplement: Supplementary file 6 — Source Data for Figure 5 [file MSB-17-e10105-s004.zip › Figure5A_sourcedata/GSEA_3017/nosigdb_stateE.GseaPreranked.1622795031093/RICHARDS_ZHONG_NATURE_2018_NPCS_UPREG.html]

Details for gene set RICHARDS\_ZHONG\_NATURE\_2018\_NPCS\_UPREG[GSEA]

|  || Dataset | state63017 |
| Phenotype | NoPhenotypeAvailable |
| Upregulated in class | na\_neg |
| GeneSet | RICHARDS\_ZHONG\_NATURE\_2018\_NPCS\_UPREG |
| Enrichment Score (ES) | -0.7684615 |
| Normalized Enrichment Score (NES) | -3.9547267 |
| Nominal p-value | 0.0 |
| FDR q-value | 0.0 |
| FWER p-Value | 0.0 |
Table: GSEA Results Summary

  

Fig 1: Enrichment plot: RICHARDS\_ZHONG\_NATURE\_2018\_NPCS\_UPREG      
 Profile of the Running ES Score & Positions of GeneSet Members on the Rank Ordered List

  

| PROBE | GENE SYMBOL | GENE\_TITLE | RANK IN GENE LIST | RANK METRIC SCORE | RUNNING ES | CORE ENRICHMENT || 1 | TAGLN2 |  |  | 32 | 0.621 | -0.0291 | No |
| 2 | WDR34 |  |  | 303 | -0.258 | -0.3432 | No |
| 3 | TGIF1 |  |  | 318 | -0.263 | -0.3561 | No |
| 4 | CNTLN |  |  | 326 | -0.264 | -0.3607 | No |
| 5 | PAICS |  |  | 336 | -0.265 | -0.3676 | No |
| 6 | MCM5 |  |  | 337 | -0.265 | -0.3640 | No |
| 7 | APOLD1 |  |  | 374 | -0.275 | -0.4025 | No |
| 8 | OIP5 |  |  | 426 | -0.291 | -0.4585 | No |
| 9 | MCM4 |  |  | 457 | -0.301 | -0.4897 | No |
| 10 | MCM3 |  |  | 560 | -0.340 | -0.6050 | No |
| 11 | HAT1 |  |  | 561 | -0.340 | -0.6003 | No |
| 12 | HMGB3 |  |  | 577 | -0.348 | -0.6132 | No |
| 13 | BTG3 |  |  | 615 | -0.365 | -0.6517 | No |
| 14 | RNASEH2A |  |  | 633 | -0.378 | -0.6665 | No |
| 15 | GINS2 |  |  | 658 | -0.399 | -0.6892 | No |
| 16 | H2AFV |  |  | 684 | -0.423 | -0.7128 | No |
| 17 | CDCA4 |  |  | 687 | -0.424 | -0.7094 | No |
| 18 | SPAG5 |  |  | 729 | -0.458 | -0.7513 | No |
| 19 | C19orf48 |  |  | 738 | -0.462 | -0.7543 | No |
| 20 | KNSTRN |  |  | 751 | -0.485 | -0.7618 | Yes |
| 21 | PRR11 |  |  | 755 | -0.488 | -0.7586 | Yes |
| 22 | CDCA3 |  |  | 759 | -0.497 | -0.7553 | Yes |
| 23 | GMNN |  |  | 766 | -0.510 | -0.7554 | Yes |
| 24 | DTYMK |  |  | 774 | -0.522 | -0.7564 | Yes |
| 25 | DEPDC1B |  |  | 779 | -0.533 | -0.7538 | Yes |
| 26 | RPA3 |  |  | 788 | -0.550 | -0.7556 | Yes |
| 27 | CDC20 |  |  | 793 | -0.562 | -0.7526 | Yes |
| 28 | HMGN2 |  |  | 795 | -0.564 | -0.7460 | Yes |
| 29 | TUBB4B |  |  | 804 | -0.573 | -0.7476 | Yes |
| 30 | CENPN |  |  | 805 | -0.574 | -0.7397 | Yes |
| 31 | MCM7 |  |  | 817 | -0.599 | -0.7444 | Yes |
| 32 | CKAP2L |  |  | 826 | -0.625 | -0.7452 | Yes |
| 33 | AURKB |  |  | 829 | -0.634 | -0.7388 | Yes |
| 34 | HELLS |  |  | 830 | -0.635 | -0.7301 | Yes |
| 35 | PCNA |  |  | 832 | -0.640 | -0.7225 | Yes |
| 36 | BUB3 |  |  | 834 | -0.641 | -0.7148 | Yes |
| 37 | CENPW |  |  | 835 | -0.643 | -0.7060 | Yes |
| 38 | CDKN3 |  |  | 836 | -0.653 | -0.6970 | Yes |
| 39 | BUB1 |  |  | 837 | -0.653 | -0.6880 | Yes |
| 40 | ITGB3BP |  |  | 838 | -0.661 | -0.6789 | Yes |
| 41 | ANP32E |  |  | 841 | -0.676 | -0.6720 | Yes |
| 42 | KIF22 |  |  | 842 | -0.678 | -0.6626 | Yes |
| 43 | BIRC5 |  |  | 843 | -0.679 | -0.6533 | Yes |
| 44 | CENPM |  |  | 844 | -0.682 | -0.6439 | Yes |
| 45 | ZWINT |  |  | 847 | -0.692 | -0.6368 | Yes |
| 46 | DHFR |  |  | 851 | -0.701 | -0.6306 | Yes |
| 47 | KIF2C |  |  | 855 | -0.721 | -0.6243 | Yes |
| 48 | NUF2 |  |  | 856 | -0.721 | -0.6143 | Yes |
| 49 | CENPK |  |  | 857 | -0.722 | -0.6044 | Yes |
| 50 | FEN1 |  |  | 862 | -0.736 | -0.5990 | Yes |
| 51 | FBXO5 |  |  | 864 | -0.742 | -0.5899 | Yes |
| 52 | CCNB2 |  |  | 865 | -0.745 | -0.5797 | Yes |
| 53 | RRM1 |  |  | 866 | -0.749 | -0.5694 | Yes |
| 54 | NDC80 |  |  | 869 | -0.762 | -0.5613 | Yes |
| 55 | ORC6 |  |  | 870 | -0.766 | -0.5507 | Yes |
| 56 | CCNA2 |  |  | 872 | -0.770 | -0.5413 | Yes |
| 57 | KIF23 |  |  | 873 | -0.772 | -0.5307 | Yes |
| 58 | MELK |  |  | 874 | -0.776 | -0.5200 | Yes |
| 59 | TMPO |  |  | 875 | -0.778 | -0.5093 | Yes |
| 60 | CKAP2 |  |  | 878 | -0.781 | -0.5009 | Yes |
| 61 | PTTG1 |  |  | 880 | -0.791 | -0.4912 | Yes |
| 62 | H2AFX |  |  | 882 | -0.799 | -0.4814 | Yes |
| 63 | KIF15 |  |  | 890 | -0.834 | -0.4782 | Yes |
| 64 | CDCA5 |  |  | 892 | -0.841 | -0.4678 | Yes |
| 65 | H2AFZ |  |  | 893 | -0.849 | -0.4561 | Yes |
| 66 | KPNA2 |  |  | 895 | -0.858 | -0.4455 | Yes |
| 67 | SMC2 |  |  | 897 | -0.867 | -0.4347 | Yes |
| 68 | PBK |  |  | 900 | -0.880 | -0.4250 | Yes |
| 69 | SMC4 |  |  | 902 | -0.886 | -0.4140 | Yes |
| 70 | DUT |  |  | 903 | -0.887 | -0.4018 | Yes |
| 71 | MIS18BP1 |  |  | 904 | -0.889 | -0.3895 | Yes |
| 72 | C21orf58 |  |  | 905 | -0.893 | -0.3773 | Yes |
| 73 | CCNB1 |  |  | 906 | -0.895 | -0.3650 | Yes |
| 74 | CKS1B |  |  | 909 | -0.912 | -0.3548 | Yes |
| 75 | HJURP |  |  | 910 | -0.921 | -0.3421 | Yes |
| 76 | CKS2 |  |  | 911 | -0.922 | -0.3294 | Yes |
| 77 | GTSE1 |  |  | 912 | -0.941 | -0.3165 | Yes |
| 78 | KIF11 |  |  | 913 | -0.957 | -0.3033 | Yes |
| 79 | CLSPN |  |  | 916 | -0.964 | -0.2924 | Yes |
| 80 | CENPU |  |  | 918 | -0.971 | -0.2802 | Yes |
| 81 | PRC1 |  |  | 919 | -0.980 | -0.2667 | Yes |
| 82 | MAD2L1 |  |  | 921 | -0.990 | -0.2543 | Yes |
| 83 | UBE2T |  |  | 922 | -0.994 | -0.2406 | Yes |
| 84 | DLGAP5 |  |  | 923 | -0.998 | -0.2269 | Yes |
| 85 | SPC25 |  |  | 924 | -1.033 | -0.2127 | Yes |
| 86 | UBE2C |  |  | 928 | -1.108 | -0.2010 | Yes |
| 87 | NUSAP1 |  |  | 929 | -1.112 | -0.1857 | Yes |
| 88 | TPX2 |  |  | 930 | -1.124 | -0.1702 | Yes |
| 89 | ASPM |  |  | 932 | -1.146 | -0.1556 | Yes |
| 90 | ATAD2 |  |  | 933 | -1.162 | -0.1396 | Yes |
| 91 | CENPF |  |  | 935 | -1.200 | -0.1243 | Yes |
| 92 | HMGB2 |  |  | 937 | -1.238 | -0.1085 | Yes |
| 93 | CDK1 |  |  | 938 | -1.248 | -0.0913 | Yes |
| 94 | RRM2 |  |  | 940 | -1.403 | -0.0732 | Yes |
| 95 | MKI67 |  |  | 941 | -1.407 | -0.0538 | Yes |
| 96 | TOP2A |  |  | 942 | -1.417 | -0.0343 | Yes |
| 97 | HIST1H4C |  |  | 946 | -2.752 | -0.0000 | Yes |
Table: GSEA details [plain text format]

  

Fig 2: RICHARDS\_ZHONG\_NATURE\_2018\_NPCS\_UPREG: Random ES distribution      
 Gene set null distribution of ES for **RICHARDS\_ZHONG\_NATURE\_2018\_NPCS\_UPREG**
